# BEX1 Promotes Imatinib-Induced Apoptosis by Binding to and Antagonizing BCL-2

**DOI:** 10.1371/journal.pone.0091782

**Published:** 2014-03-13

**Authors:** Qian Xiao, Yeting Hu, Yue Liu, Zhanhuai Wang, Haitao Geng, Lifeng Hu, Dengyong Xu, Ke Wang, Lei Zheng, Shu Zheng, Kefeng Ding

**Affiliations:** 1 The Key Laboratory of Cancer Prevention and Intervention of China National Ministry of Education, The Key Laboratory of Molecular Biology in Medical Sciences of Zhejiang Province, Cancer Institute, Hangzhou, Zhejiang, China; 2 The Second Affiliated Hospital of Zhejiang University School of Medicine, Hangzhou, Zhejiang, China; 3 Department of Oncology and Department of Surgery, The Sidney Kimmel Comprehensive Cancer Center at Johns Hopkins, Johns Hopkins University School of Medicine, Baltimore, Maryland, United States of America; Innsbruck Medical University, Austria

## Abstract

An enhanced anti-apoptotic capacity of tumor cells plays an important role in the process of breakpoint cluster region/Abelson tyrosine kinase gene (BCR/ABL)-independent imatinib resistance. We have previously demonstrated that brain expressed X-linked 1 (BEX1) was silenced in secondary imatinib-resistant K562 cells and that re-expression of BEX1 can restore imatinib sensitivity resulting in the induction of apoptosis. However, the mechanism by which BEX1 executes its pro-apoptotic function remains unknown. We identified B-cell lymphoma 2 (BCL-2) as a BEX1-interacting protein using a yeast two-hybrid screen. The interaction between BEX1 and BCL-2 was subsequently confirmed by co-immunoprecipitation assays. Like BCL-2, BEX1 was localized to the mitochondria. The region between 33K and 64Q on BEX1 is important for its localization to the mitochondria and its ability to interact with BCL-2. Additionally, we found that this region is essential for BEX1-regulated imatinib-induced apoptosis. Furthermore, we demonstrated that the interaction between BCL-2 and BEX1 promotes imatinib-induced apoptosis by suppressing the formation of anti-apoptotic BCL-2/BCL-2-associated X protein (BAX) heterodimers. Our results revealed an interaction between BEX1 and BCL-2 and a novel mechanism of imatinib resistance mediated by the BEX1/BCL-2 pathway.

## Introduction

Targeted inhibition of tyrosine kinases with imatinib (imatinib mesylate) is now a front line therapy for patients with chronic myelogenous leukemia (CML) or gastrointestinal stromal tumors (GISTs). However, nearly 33% of all CML patients and 50% of all GISTs patients show disease progression during imatinib therapy due to the development of secondary resistance [1], [2]. Several mechanisms have been proposed to account for this resistance, including breakpoint cluster region/Abelson tyrosine kinase gene (BCR/ABL)-dependent or BCR/ABL-independent mechanisms [2], [3]. BCR/ABL-dependent resistance mechanisms involve BCR/ABL mutations, which alter the binding affinity of imatinib to the BCR/ABL tyrosine kinase, and amplification, which leads to increased expression of the BCR/ABL kinase [4], [5]. BCR/ABL-independent resistance mechanisms include processes that affect drug delivery [5], [6]. Additionally, enhanced suppression of apoptosis in tumor cells plays an important role in the process of BCR/ABL-independent imatinib resistance [7]. Burchert et al. showed that activation of the anti-apoptotic PI3K/AKT/mTOR pathway occurs during the early stages of imatinib resistance, and inhibiting PI3K/AKT activation blocked the development of imatinib resistance [8]. Activation of the JAK-2/STAT-5 pathway and alterations in P53 were also reported in imatinib-resistant CML patients [9], [10].

Tumor cells acquire resistance to apoptosis through various mechanisms that interfere at different stages of apoptosis signaling. One mechanism of resistance is the over-expression of anti-apoptotic genes. In both *in vitro* and *in vivo* models, B-cell lymphoma 2 (BCL-2) expression confers resistance to many types of chemotherapeutic drugs [11], [12], [13]. Besides over-expression of anti-apoptotic genes, tumors can acquire apoptosis resistance by downregulating or genetically altering pro-apoptotic molecules. For instance, inhibition of BCL-2 interacting mediator (BIM) expression by RNAi can inhibit the killing effect of imatinib on GISTs cells and BCR/ABL+ tumor cells [14], [15].

In our previous study, we established an imatinib-resistant K562 cell line (KR) by progressively increasing the dose of imatinib in the culture medium [16]. Interestingly, resistance to imatinib in the KR cells is through a BCR/ABL-independent manner. BCR/ABL mRNA and protein expression in KR cells was not significantly increased compared to the original K562 cells, and no mutation was found in the exons of the BCR/ABL locus. When analyzing the gene expression profile of KR cells, we discovered that the human brain expressed X-linked 1 (BEX1) gene, which is involved in apoptosis, was silenced. Re-expression of BEX1 in KR cells restored drug sensitivity by inducing apoptosis. BEX1 belongs to a family of six genes with a wide tissue distribution in the human body, including the brain, pancreas, testis, and ovary [17], [18]. Little is known about the function of the BEX1 protein, which is only 128 amino acids long. Foltz et al. reported that BEX1 was silenced in malignant gliomas as a result of extensive promoter hypermethylation [19]. Furthermore, re-expression of BEX1 resulted in a significant suppression of tumor growth and apoptosis induction in response to camptothecin treatment.

Here, to better understand the role of BEX1 in imatinib-induced apoptosis, we sought to identify BEX1-interacting proteins using a yeast two-hybrid screen. The cDNA library used for this screen originated from imatinib-resistant K562 cells that overexpress BEX1 to overcome the imatinib resistance. BCL-2 was subsequently identified as a potential BEX1 binding partner. The interaction between BEX1 and BCL-2 appears to be important for the induction of apoptosis in response to imatinib. These findings have provided new evidence for the mechanism of BEX1/BCL-2-mediated anti-apoptosis process and imatinib resistance.

## Materials and Methods

### Plasmid Construction

The general protocol for plasmid construction was previously described [16]. All empty vectors were purchased from Takara Biomedical Technology (Beijing, China). All primers were listed in Table S1. For yeast two-hybrid screening, BEX1 was cloned into the pGBKT7 plasmid. For co-immunoprecipitation experiments, several BEX1 truncated mutants were amplified and cloned into the pCMV-HA vector using BEX1 or a synthesized DNA fragment as a template. For the BEX1 localization assay, BEX1 was integrated into either the pEGFP-C1 or pEGFP-N1 plasmid. All of the recombinant plasmids were identified and confirmed by DNA sequencing.

### Cell Culture and Imatinib Treatment

All cells were maintained in medium containing penicillin and streptomycin at 37°C with 5% CO_2_. HEK293 cells obtained from the American Type Culture Collection (Rockville, MD, USA) were cultured in Dulbecco’s Modified Eagle Medium (DMEM) media containing 10% fetal bovine serum (FBS). The KR cell line, which is an imatinib-resistant cell line, was established using K562 cells (American Type Culture Collection) as previously described [16]. Additionally, the KR/BEX1 cell line was established by stably expressing BEX1 in KR cells as previously described [16]. KR and KR/BEX1 cells were cultured in RPMI 1640 media (Jinuo Biotech, Hangzhou, China) supplemented with 10% FBS. To induce apoptosis, 48 hours after transfection (see below) the cells were treated with 2 µM imatinib (Selleck Chemicals, Houston, TX, USA) with or without 0.2 µM ABT-737 (BH3 mimeticse) (Selleck Chemicals, Houston, TX, USA) for 24 hours.

### Cell Transfection

Transfection of HEK293 cells was performed using PolyJet (Mingrui Biotech, Shanghai, China) according to the manufacturer’s protocol. For KR cell transfection, PolyJet was used according to a modified protocol. Briefly, the Polyjet/DNA complex was diluted and mixed at a ratio of 4∶1 (µl Polyjet: µg DNA) in serum-free DMEM with high glucose (4.5 g/l). Next, the K562 cells were harvested and then gently resuspended in the liposome-DNA complex followed by incubation at 37°C for 20 minutes. Following the incubation, pre-warmed fresh complete cell growth medium was added to the cells, and the cells were plated in cell culture dishes.

### KR/BEX1 cDNA Library Construction and Yeast Two-hybrid Screening

The cDNA library for the yeast two-hybrid screening was made from the KR/BEX1 cells. KR/BEX1 cDNA library construction and yeast two-hybrid screening were performed using Matchmaker Library Construction & Screening Kits from Takara Biomedical Technology according to the manufacturer’s instructions. Briefly, total RNA was extracted from KR/BEX1 cells, reversed transcribed using a random CDS III/6 primer and SMART III primer, and amplified into double stranded (ds) cDNA using a pair of specific primers (Table S1). The amplified ds cDNA, pGADT7-Rec and pGBKT7-BEX1, as bait, were co-transformed into competent AH109 yeast cells. A cDNA library was constructed by fusing cDNA fragments with the Sma I linearized pGADT7-Rec plasmid through a recombination-mediated cloning mechanism in yeast. Positive clones were selected by using solid dropout SD/-Ade/-His/-Leu/-Trp culture media after incubation for 3–5 days at 30°C. Plasmid DNA was extracted from positive clones and was co-transfected along with pGBKT7-BEX1 into competent AH109 yeast cells to verify positive protein-protein interactions using solid dropout SD/-Ade/-His/-Leu/-Trp culture media. Once verified, the cDNA inserts in the positive plasmids were sequenced. In the yeast two-hybrid assay, the interaction between the SV40 large T antigen and the P53 protein was used as a positive control, and the absence of an interaction between the SV40 large T antigen and the human laminin C protein was used as a negative control.

### Subcellular Fractionation and Analysis

Mitochondria-enriched fractionation was performed using the Mitochondria Isolation Kit (Thermo Scientific, Pittsburgh, PA, USA) according to the manufacturer’s instructions. The fractions were examined by Western blot analysis using anti-green fluorescent protein (GFP) (SC-9996, Santa Cruz Biotechnology, Santa Cruz, CA, USA), cytochrome c oxidase (COX) IV (#4850, Cell Signaling Technology, Beverly, MA, USA) and glyceraldehyde-3-phosphate dehydrogenase (GAPDH, KC-5G4, Kangchen Biotechnology, Shanghai, China) antibodies.

### Live Cell Fluorescent Microscopy

KR cells transfected with the pEGFP-C1/BEX1 or pEGFP-N1/BEX1 plasmid were grown on glass bottom dishes (GWST-3522, WillCo Wells, Amsterdam, The Netherlands). Forty-eight hours after transfection, the cells were cultured for 45 minutes in an incubator with 200 nM MitoTracker Red CMXRos (M-7512, Life Technology, Carlsbad, CA, USA) diluted in complete culture medium. Then, the cells were washed three times with warm Hank’s balanced salt solution and cultured in fresh pre-warmed medium. Microscopy observations were performed using a Zeiss LSM 710 laser-scanning confocal imaging system (Carl Zeiss AG, Oberkochen, Germany). GFP fluorescence was detected between 505 nm and 550 nm with excitation at 488 nm. MitoTracker staining was detected between 585 nm and 615 nm with excitation at 568 nm.

### Co-immunoprecipitation and Western Blot Analysis

As previously described [16], cell lysates were incubated at 4°C overnight with 2 µg of rabbit anti-BCL-2 antibody (#1017-1, Epitomics, Hangzhou, China), rabbit anti-hemagglutinin (HA) antibody (#3724, Cell Signaling Technology, Beverly, MA, USA), or an isotype control rabbit IgG antibody (A7016, Beyotime, Nanjing, China). The samples were subsequently precipitated with protein A/G-agarose beads (SC-2003, Santa Cruz Biotechnology, Santa Cruz, CA, USA) at 4°C for 2 hours. The beads were washed three times in 1% 3-[(3-cholamidopropyl) dimethylammonio]-1-propanesulfonate (CHAPS), and bound proteins were eluted.

Western blotting was performed as described previously [16] using mouse anti-BCL-2 (#551097) from BD Pharmingen (San Jose, CA, USA), mouse anti-HA-tag (#2367), rabbit anti-BCL-2 associated X protein (BAX) (#2772), rabbit anti-pBCL-2 (ser70) (#2827), mouse anti-caspase-3 (#9668), rabbit anti-cleaved caspase-3 (#9664), and rabbit anti-cleaved caspase-9 (#9501) antibodies, all purchased from Cell Signaling Technology. For protein standardization, we used mouse anti-GAPDH (KC-5G4, Kangchen Biotechnology, Shanghai, China).

### RNA Interference

Validated short hairpin RNA directed against BAX and control short hairpin RNA were obtained from Genechem (Shanghai, China). Transfections were performed using PolyJet according to the modified protocol described above. The efficiency of the BAX short hairpin RNA was determined by Western blot analysis using anti-BAX antibodies at 48 hours.

### Flow Cytometry Assay

Cells were plated at a density of 1×10^6^/well in 6-well plates 24 hours before the induction of apoptosis. After treatment with 2 µM imatinib with or without 0.2 µM ABT-737 for 24 hours, the cells were harvested and double-stained with FITC-conjugated annexinV and propidium iodide (PI) using an AnnexinV–FITC Apoptosis Detection Kit (SouthernBiotech, Birmingham, AL, USA) according to the manufacturer’s protocol. Then, the cells were analyzed using a BD FACSCalibur Flow Cytometer (BD Biosciences, San Jose, CA, USA) within 1 hour of staining. Apoptotic cells were defined as annexinV–FITC positive and PI negative cells. All experiments were performed in triplicate and were independently repeated three times.

### Statistical Analysis

The flow cytometry data were presented as the means ± standard error of the mean (SEM). These data were analyzed by a two-tail unpaired Student’s t-test. P<0.05 indicated statistical significance.

## Results

### Identification and Validation of BCL-2 as a BEX1-binding Partner

To gain further insight into the function of BEX1, we screened BEX1-interacting proteins in a yeast two-hybrid system based on the eukaryotic transcription factor GAL4 using full-length BEX1 as the bait. From this screen, we identified 13 positive clones. Among the clones identified, one clone corresponded to the complete coding sequence of BCL-2, an important molecule involved in apoptosis regulation. The interaction between full-length BEX1 and BCL-2 proteins was further verified in the yeast two-hybrid assay (Table 1).

**Table 1:** Identification of BCL-2 as an interaction partner for BEX1 by a yeast two-hybrid screen.

BD plasmid	AD plasmid	Growth on -Ade-His
pGBKT7/BEX1	–	–
–	pGADT7-Rec/BCL-2	–
pGBKT7/BEX1	pGADT7-Rec/BCL-2	+
pGBKT7/P53	pGADT7-Rec/SV40-T	+
pGBKT7/LAM	pGADT7-Rec/SV40-T	–

BD, binding domain; AD, activation domain; LAM, laminin C; SV40-T, SV40 large T; P53, protein 53.

**Figure 1:**
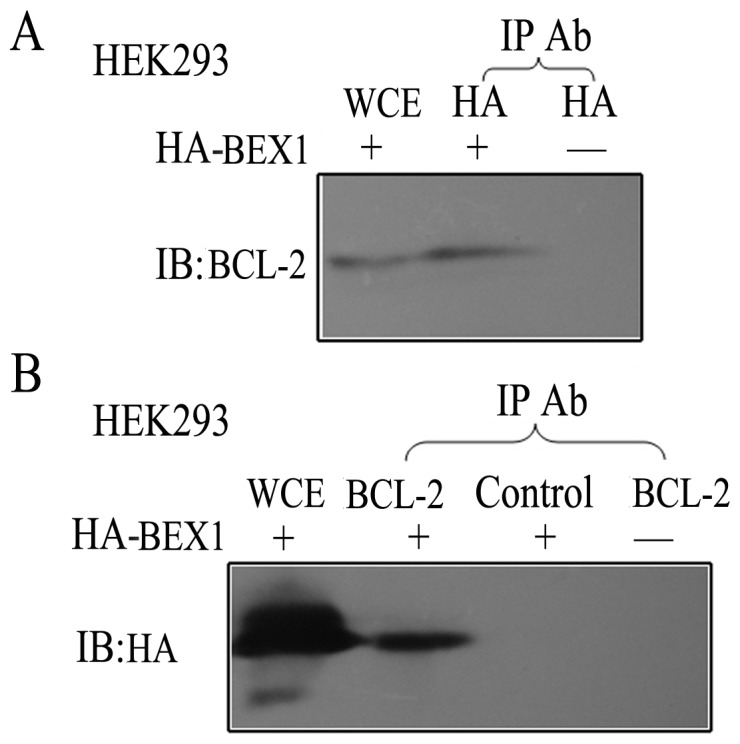
BEX1 interacts with the anti-apoptotic protein BCL-2. “+” and “−” refers to whether or not cells were transfected with HA-BEX1. If the cells were not transfected with HA-BEX1, an empty vector pCMV-HA was used for transfection. “Control” refers to the rabbit IgG isotype control. A, BEX1 was immunoprecipitated using an anti-HA antibody, and the co-immunoprecipitate (co-IP) was analyzed by immunoblotting (IB) with an anti-BCL-2 antibody. The intrinsic BCL-2 was monitored in the whole-cell extract (WCE). B, BCL-2 was immunoprecipitated using an anti-BCL-2 antibody or isotype control rabbit IgG, and the co-IP was analyzed by IB with an anti-HA antibody. The presence of HA-BEX1 was also monitored in the WCE. The lower band was a non-specific signal.

To confirm the yeast two-hybrid results, we examined the ability of BEX1 and BCL-2 to interact in HEK293 cells transfected with the pCMV-HA/BEX1 plasmid that expressed an exogenous HA-tagged BEX1 protein. Both the anti-BCL-2 antibody and anti-HA antibody, but not the isotype control rabbit IgG, were able to co-immunoprecipitate both the BCL-2 and HA-BEX1 proteins (Figure 1). Therefore, these results confirmed an interaction exists between BEX1 and BCL-2.

### BEX1 Partially Localizes to Mitochondria

**Figure 2:**
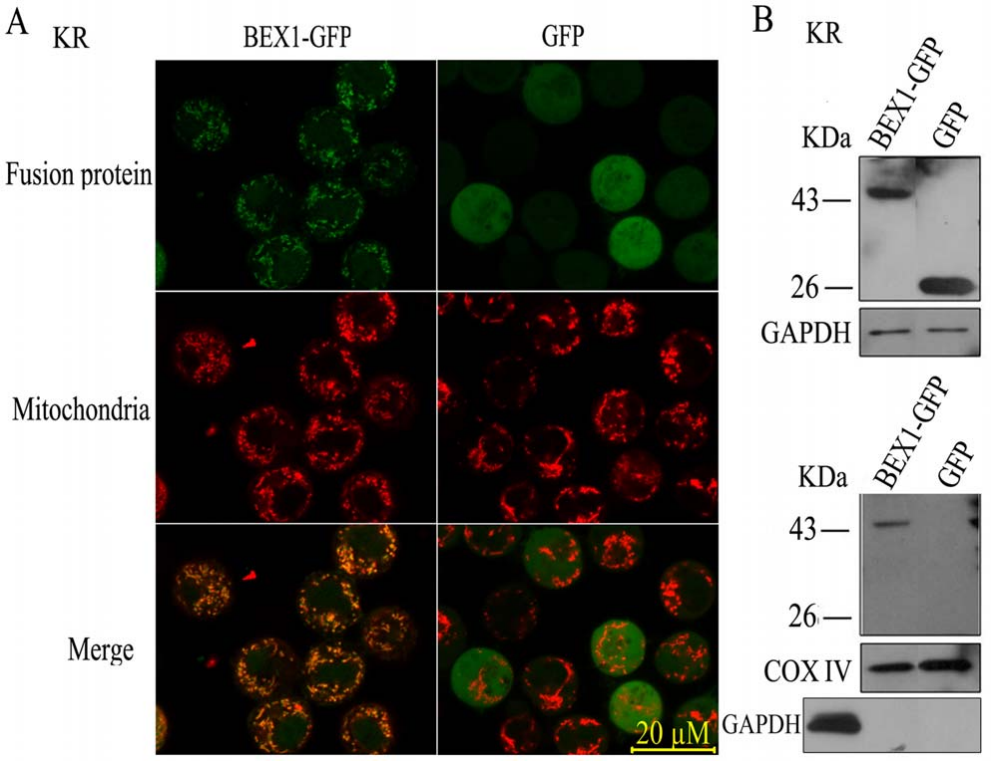
BEX1 localizes to the mitochondria. A, Fluorescence of live KR cells expressing BEX1-GFP or an empty vector control (GFP). Cells were visualized for GFP (top), Mitotracker (middle), or merged images (bottom). B, Biochemical fractionation. WCE prepared from KR cells expressing BEX1-GFP or an empty vector control (GFP) were separated into cytoplasmic (top) and mitochondrial (bottom) fractions and then immunoblotted for GFP, GAPDH (a cytoplasmic protein), or cyclooxygenase IV (COX IV, a mitochondria marker). Cross contamination of mitochondrial fractions in the cytoplasmic fractions was excluded by immunoblotting with GAPDH.

BEX1 is reported to primarily localize to the cytoplasm in all types of cells and to a lesser extent in the nucleus of breast cancer cells [20], [21]. Because BCL-2 is localized to the mitochondria, the interaction between BEX1 and BCL-2 suggests that BEX1 may co-localize with BCL-2 in the mitochondria. To test this, we examined the subcellular localization of the BEX1 protein in HEK293 and KR cell lines that were transfected with plasmids expressing BEX1 tagged with GFP at the C-terminus (BEX1-GFP) using confocal microscopy. The BEX1-GFP fusion proteins were localized to the mitochondria marked by the MitoTracker Red CMXRos in both KR cells (Figure 2A) and in HEK293 cells (not shown). Similarly, expression of BEX1 tagged with GFP at the N-terminus (GFP-BEX1) in KR cells overlapped with mitochondrial staining (Figure S1).

To further confirm the localization of BEX1, we performed biochemical fractionation of mitochondrial proteins from KR cells transfected with the fluorescent plasmids. The results showed that BEX1 was enriched in the fraction that contained the mitochondria and co-fractionated with the mitochondrial marker protein COX IV (Figure 2B).

### Residues 33K-64Q on BEX1 are Important for its Interaction with BCL-2 and Localization to the Mitochondria

**Figure 3:**
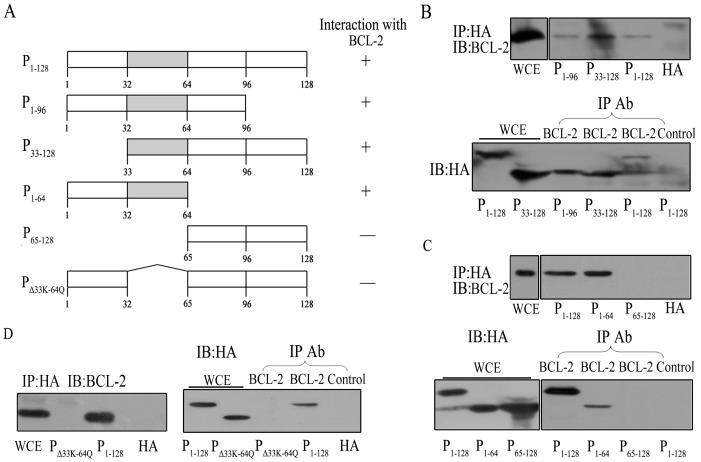
Residues 33K-64Q are important for the interaction between BEX1 and BCL-2. A, Schematic representations of HA-tagged BEX1 deletion mutants and their ability to bind to BCL-2. B–D, HEK293 cells were transfected with plasmids expressing HA-BEX1 and HA-tagged BEX1 deletion mutants. BEX1 was immunoprecipitated using an anti-HA antibody, and the co-immunoprecipitate (co-IP) was analyzed by immunoblotting with an anti-BCL-2 antibody. BCL-2 was immunoprecipitated using an anti-BCL-2 antibody or isotype control rabbit IgG, and the co-IP was analyzed by IB with an anti-HA antibody. The presence of HA-BEX1, HA-tagged BEX1 deletion mutants and BCL-2 were monitored in the WCE.

Next, we sought to determine which regions of BEX1 are important for its interaction with BCL-2. To this end, several BEX1 truncated mutants were created and cloned into the pCMV-HA vector. The resultant plasmids expressed HA-tagged BEX1 proteins and were transfected into HEK293 cells. Lysates of these cells were subsequently precipitated using anti-BCL-2 antibodies for endogenous BCL-2 or by anti-HA antibodies for HA-tagged BEX1 proteins. The results showed that BCL-2 was only able to be co-immunoprecipitated with full-length BEX1 (P_1–128_) or BEX1 truncated mutants containing residues 1–64 or 1–96. The truncated mutant containing residues 65–128 was not able to be co-immunoprecipitated with BCL-2, suggesting that the 64 amino acids at the N-terminal region of BEX1 are important for its interaction with BCL-2 (Figure 3). BCL-2 was also able to be co-immunoprecipitated with the BEX1 mutant (P_33–128_), which is missing the first 32 amino acids in the N-terminus, further narrowing down the BCL-2 binding region to between residues 33K and 64Q. To further confirm that the region in BEX1 between residues 33K and 64Q is important for its interaction with BCL-2, a BEX1 mutant with a deletion of residues 33K-64Q was made and was unable to co-immunoprecipitate with BCL-2.

**Figure 4:**
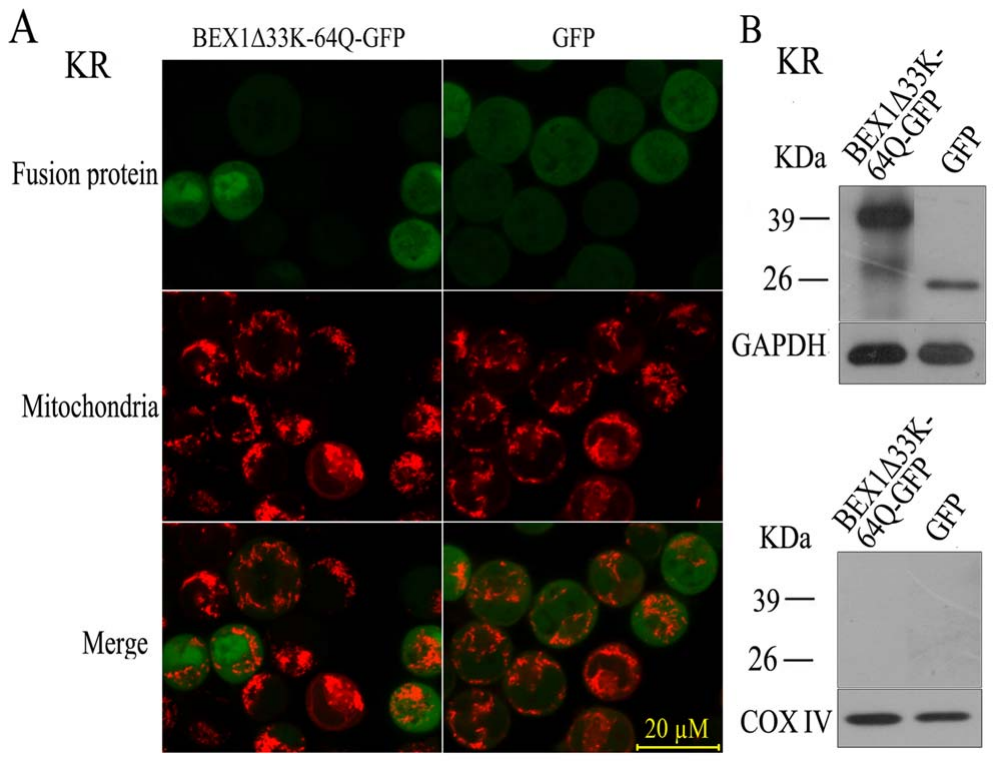
BEX1 fails to localize to the mitochondria without residues 33K-64Q. A. Fluorescence of live KR cells expressing BEX1Δ33K-64Q-GFP or an empty vector control (GFP). Cells were visualized for GFP (top), Mitotracker (middle), or merged images (bottom). B, Biochemical fractionation. WCE prepared from KR cells expressing BEX1Δ33K-64Q-GFP or an empty vector control (GFP) were separated into cytoplasmic (top) and mitochondrial (bottom) fractions as in Figure 2 and then immunoblotted for GFP, GAPDH, or COX IV. The bands between 39 kDa and 26 kDa were non-specific signals.

Interestingly, we found that BEX1_Δ33K-64Q_-GFP, the BEX1 mutant with a deletion of residues 33K-64Q tagged with GFP at the C-terminus, failed to localize to the mitochondria (Figure 4A). Biochemical fractionation of mitochondrial proteins from KR cells transfected with the plasmid expressing BEX1_Δ33K-64Q_ did not find this mutant BEX1 protein in the mitochondrial fraction (Figure 4B). The GFP-BEX1_Δ33K-64Q_ fusion protein with the GFP in the N-terminus did not localize to the mitochondria either (Figure S2). These results suggest that residues 33K-64Q on BEX1 are important for its localization to the mitochondria. It is possible that without the region between residues 33K and 64Q, BEX1 would not be able to interact with BCL-2 because it is unable to localize to the mitochondria. Alternatively, if its interaction with BCL-2 were important for the mitochondrial localization of BEX1, without the region between residues 33K and 64Q, BEX1 would fail to localize to the mitochondria because it would be unable to bind BCL-2.

### BEX1 Promotes Apoptosis by Interfering with BCL-2 Phosphorylation and its Subsequent Heterodimerization with BAX

**Figure 5:**
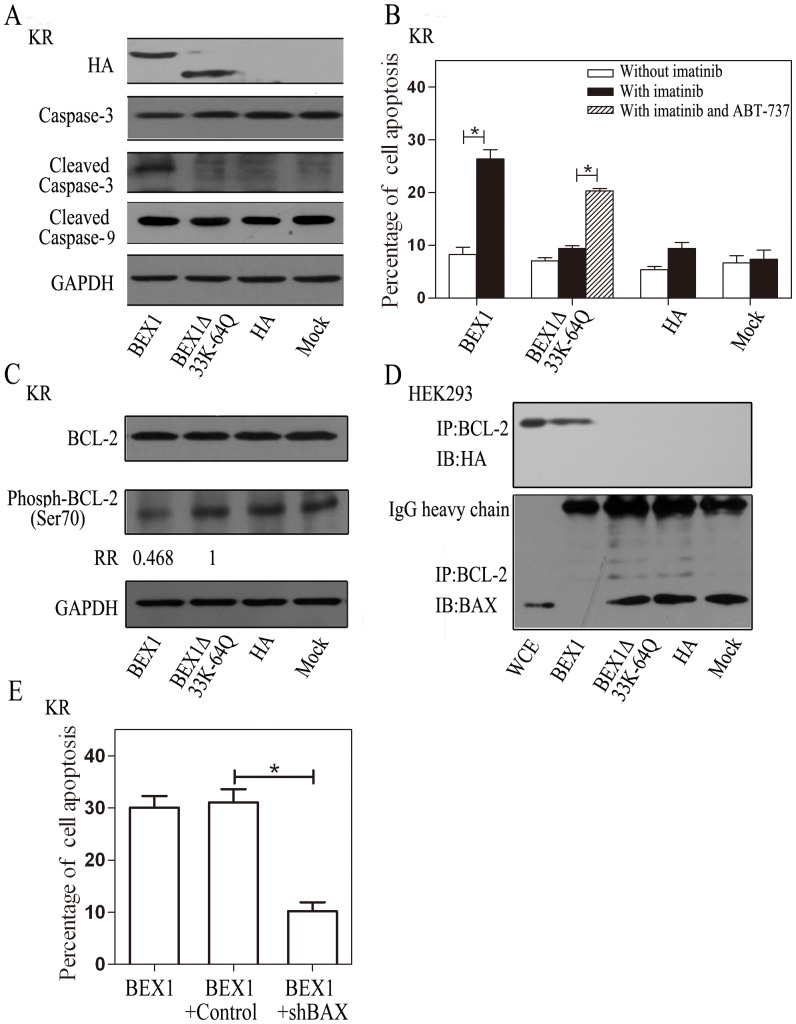
BEX1 promotes apoptosis by interfering with BCL-2 phosphorylation and heterodimerizing with BAX. KR cells or HEK293 cells were transfected with plasmids expressing HA-BEX1, HA-BEX1Δ33K-64Q, empty vector control (Panel A, B, C, D), or co-transfected with HA-BEX1 and shRNA targeting BAX (shBAX) (Panel E). Forty-eight hours after transfection, 2 µM imatinib (Panel A, C and E) with or without 0.2 µM ABT-737 (Panel B) was added to the culture medium for 24 hours. A, Caspase-3, cleaved caspase-3, and cleaved caspase-9 from KR cells were detected by immunoblotting analysis after the induction of apoptosis. GAPDH was used as a loading control. B, Following the induction of apoptosis, KR cells were stained with annexin V conjugated FITC and PI, and then examined by flow cytometry. Error bars represent the means ± SEM, *p<0.05. C, BCL-2, Phospho-BCL-2 (ser70), and GAPDH were analyzed by immunoblotting. Phospho-BCL-2 (ser70) bands were quantified by the Image J software and are shown as the relative ratio (RR) between HA-BEX1 and HA-BEX1Δ33K-64Q transfected cells. D, BCL-2 was immunoprecipitated with an anti-BCL-2 antibody, and the co-IP was analyzed by immunoblotting with an anti-HA antibody or an anti-BAX antibody. The upper bands on the anti-BAX blot were the IgG heavy chain. E, KR cells were co-transfected with the shRNA for BAX knockdown (shBAX) or the control shRNA (control) with HA-BEX1. After the induction of apoptosis, KR cells were double stained with annexin V conjugated FITC and PI as in panel B, and then examined by flow cytometry. Error bars represent the means ± SEM, *p<0.05.

Previous studies have demonstrated that BEX1 activates caspase-3 to induce cell apoptosis [16]. Therefore, we hypothesized that BEX1 mediates imatinib-induced apoptosis through an apoptotic pathway involving BCL-2. To test this hypothesis, we transfected KR cells with BEX1_Δ33K-64Q_ and determined whether this plasmid was able to reverse the resistance of these cells to imatinib treatment and promote imatinib-induced apoptosis. The cleavage of caspase-3 was used as a marker of apoptosis. Twenty-four hours following treatment with imatinib, there was no apparent increase in the cleavage of caspase-3 in the KR cells transfected with BEX1_Δ33K-64Q_ (Figure 5A); whereas, as reported previously [16], wild-type BEX1 induced the cleavage of caspase-3 in the KR cells. Consistent with our previous study [16], there was no apparent increase in the expression of cleaved caspase-9 in BEX1-overexpressing KR cells after 24 hours of imatinib treatment. Unlike wild-type BEX1, BEX1_Δ33K-64Q_ failed to induce apoptosis in the presence of imatinib, and thus, failed to reverse the resistance of the KR cells to imatinib treatment (Figure 5B). We tested BEX1Δ33K-64Q overexpressing KR cells treated with 2 µM imatinib with or without 0.2 µM ABT-737 (BH3 mimeticse) for 24 hours. Apoptosis was induced significantly (p<0.05, Figure 5B) by ABT-737 in KR cells expressing mutated BEX1. This result suggested that the deficiency in BEX1 could be bypassed by treating the cells with the BH3 mimetics to directly inhibit BCL-2. These results suggest that residues 33K-64Q on BEX1, a region important for its interaction with BCL-2, is critical for imatinib-induced apoptosis and the sensitivity of K562 cells to imatinib treatment.

To further demonstrate that the function of BEX1 in imatinib-induced apoptosis involves BCL-2, we determined if the expression of BEX1 influenced BCL-2 expression and phosphorylation, which are two mechanisms that regulate the function of BCL-2 in apoptosis [22], [23]. Decreased expression of BCL-2 or phosphorylation is known to suppress the anti-apoptotic function of BCL-2. The expression of BCL-2 was not significantly changed in the KR cells transfected with wild-type BEX1 in the presence of imatinib compared to the cells transfected with BEX1_Δ33K-64Q_ (Figure 5C). Conversely, there was an approximately 50% reduction in the expression of ser70 phosphorylated BCL-2 in the KR cells with wild-type BEX1 compared to BEX1_Δ33K-64Q_ cells. This result suggests that the anti-apoptotic function of BCL-2 is suppressed when BEX1 is expressed to promote imatinib-induced apoptosis.

It is well characterized that BCL-2 mediates its anti-apoptotic function, in part, by heterodimerizing with BAX to inhibit the pro-apoptotic activity of BAX [24], [25]. BAX dissociates from BCL-2 during the onset of apoptosis. Therefore, we investigated whether or not BEX1-induced apoptosis also involves the dissociation of BAX from BCL-2. Wild-type BEX1, but not BEX1_Δ33K-64Q_, inhibited the association between BCL-2 and BAX (Figure 5D), which suggests that the region important for BAX and BCL-2 interaction is also critical for the dissociation of BAX from BCL-2. To further confirm that BEX regulates apoptosis through BAX/BCL-2, we co-transfected BAX shRNA with BEX1 cDNA into KR cells (Figure S3). We found that knockdown of BAX suppresses imatinib-induced apoptosis in BEX1 overexpressing KR cells (p<0.05, Figure 5E and Figure S4). Taken together, our data suggest that the interaction between BEX1 and BCL-2 mediates the pro-apoptotic function of BEX1.

## Discussion

In this study, we revealed that BEX1 as a novel binding partner of BCL-2. Like BCL-2, BEX1 also localizes to the mitochondria. This study also revealed that the region between amino acids 33K and 64Q on BEX1 is essential its localization to the mitochondria and interaction with BCL-2. Additionally, we found that the interaction between BCL-2 and BEX1 is essential for imatinib-induced apoptosis. Moreover, we found that binding of BCL-2 to BEX1 interferes with ser70 phosphorylation of BCL-2, which prevents BCL-2 from heterodimerizing with BAX. Thus, this study has provided a new mechanism that accounts for imatinib resistance and BCL-2-mediated regulation of apoptosis.

BEX1 is over-expressed in a subset of primary breast cancers [26] and has been shown to prevent breast cancer cells from undergoing apoptosis [27]. Downregulation of BEX1 induces apoptosis and sensitizes breast cancer cells to pro-apoptotic reagents including ceramide and doxorubicin [26]. However, as described above, Foltz et al. reported that BEX1 was downregulated in malignant glioma, and re-expression of BEX1 resulted in a significant suppression of tumor growth and induced apoptosis in response to camptochecin [19]. Thus, BEX1 appears to have different functions in different tumor types. Nonetheless, both studies suggested a role for BEX1 in regulating apoptosis and mechanisms of drug resistance. It is possible that the expression level of BEX1 is tightly regulated, and either an overexpression or significant downregulation of BEX1 expression could lead to misregulation of BEX1 and drug resistance in the tumor cells. Our studies have further supported the pro-apoptotic function of BEX1. We have shown previously [16], as well as in this study, that downregulation of BEX1 is associated with imatinib resistance and an anti-apoptotic capacity of imatinib-resistant K562 cells, whereas re-expression of BEX1 sensitized the cells to imatinib-induced apoptosis.

In this study, we further explored the mechanism of BEX1-mediated apoptosis by delineating the interaction between BEX1 and BCL-2. Although it is still not clear if BEX1 binds BCL-2 directly or indirectly, this study used three independent assays, including a yeast two-hybrid analysis, a co-immunoprecipitation assay, and mitochondrial co-staining analysis to support a tight association between these two proteins. In an attempt to identify the region on BEX1 that is important for its interaction with BCL-2, we found that the region between 33K and 64Q was critical for the localization of BEX1 to the mitochondria. It is possible that BEX1, without this region, fails to localize to the mitochondria, and thus is unable to interact with BCL-2. Alternatively, without this region, the inability to interact with BCL-2 could lead to the dissociation of BEX1 from mitochondria. Further exploration of the mechanism underlying the mitochondrial localization of BEX1 is warranted.

Our study suggests that the essential role of BEX1 in the induction of apoptosis in response to imatinib treatment is mediated by BCL-2. BCL-2 has emerged as a negative regulator of apoptosis when expressed at relatively high levels in malignant diseases [28] and has been shown to be a mediator of drug resistance [29]. BCL-2 phosphorylation is a consequence of the dynamic balance between a variety of kinases and phosphatases and is thought to be important to the anti-apoptotic function of BCL-2. It is also believed that BCL-2 suppresses apoptosis by sequestering pro-apoptotic proteins, such as BAX [25], [30]. Therefore, it is conceivable that, similar to BIM [24], [31], BEX1 may release pro-apoptotic proteins such as BAX by sequestering BCL-2. Our data showing that expression of BEX1 downregulates the phosphorylation of BCL-2 at ser70 suggests another mechanism by which BEX1 antagonizes the function of BCL-2. Alternatively, however, the binding of BEX1 to BCL-2 may physically block ser70 phosphorylation. Nonetheless, the mechanism by which BEX1 regulates BCL-2 phosphorylation, inhibits the formation of the BCL-2/BAX complex, and whether or not it can also inhibit the interaction between BCL-2 and other pro-apoptotic proteins remain to be explored.

There was no obvious activation of caspase-9 in BEX1-overexpressing KR cells following 24 hours of imatinib treatment. This result is consistent with our previous report showing no induction of caspase-9 activation in KR/BEX1 cells treated with imatinib. However, such results do not exclude the involvement of the intrinsic/mitochondrial pathway of apoptosis. BEX1 may activate apoptosis by inhibiting an inhibitor of apoptosis proteins (IAP). Burke SP et al. showed that cIAP1 prevents the activation of procaspase-3 but has no effect on the processing of procaspase-9. Additionally, they showed that X-linked IAP (XIAP) has no effect on procaspase-9 processing but is a potent inhibitor of procaspase-3 activation. However, they found that cIAP1 cooperatively inhibits procaspase-3 activation by the caspase-9 apoptosome [32]. Thus, it will be interesting to examine the role of BEX1 in the IAP pathways.

In summary, our study discovered an interaction between BEX1 and BCL-2 and subsequently revealed a novel mechanism of imatinib resistance that is mediated by the BEX1/BCL-2 pathway. Future studies are warranted to investigate whether this mechanism of imatinib resistance is present in patients treated with imatinib and whether the same mechanism mediates resistance to other cancer therapeutics. Such studies will provide a rationale for the utilization of innovative therapies that target the BEX1/BCL-2 pathway to enhance the sensitivity of patients to imatinib and other cancer treatments.

## Supporting Information

Figure S1 **GFP-BEX1 localizes to the mitochondria.** A, Fluorescence of live KR cells expressing GFP-BEX1. Cells were visualized for GFP (top), Mitotracker (middle), or merged images (bottom). B, Biochemical fractionation. WCE prepared from KR cells expressing BEX1-GFP, GFP-BEX1 or an empty vector control (GFP) were separated into cytoplasmic (top) and mitochondrial (bottom) fractions and then immunoblotted for GFP, GAPDH, or COX IV. BEX1-GFP showed a better localization to mitochondria (Figure 2A) than GFP-BEX1. Consistently, biochemical fractionation also showed that more BEX1-GFP localizes to the mitochondrial fraction than GFP-BEX1. Also, BEX1-GFP had a larger molecular weight than GFP-BEX1. Although the exact reason for the difference in molecular weight between BEX1-GFP and GFP-BEX1 is not known, one possible explanation is that the GFP-BEX1 visualized on the Western blot may be a degraded form of the protein. This may also explain why less GFP-BEX1 is localized to the mitochondria.
(TIF)

Figure S2 **GFP-BEX1Δ33K-64Q fails to localize to the mitochondria without residues 33K-64Q.** A. Fluorescence of live KR cells expressing GFP-BEX1Δ33K-64Q. Cells were visualized for GFP (top), Mitotracker (middle), or merged images (bottom). B, Biochemical fractionation. WCE prepared from KR cells expressing GFP-BEX1Δ33K-64Q or an empty vector control (GFP) were separated into cytoplasmic (top) and mitochondrial (bottom) fractions and then immunoblotted for GFP, GAPDH, or COX IV. The bands between 39 kDa and 26 kDa were non-specific signals.
(TIF)

Figure S3 **BAX expression in KR cells following shRNA knockdown of BAX.** BAX expression was quantified using ImageJ software and was shown as the relative ratio (RR) compared to control shRNA (control) transfected cells. The knockdown efficiency of BAX expression was achieved using shRNA targeting BAX (shBAX), which decreased expression by approximately 50%.
(TIF)

Figure S4 **Knockdown of BAX suppresses imatinib-induced apoptosis in BEX1 overexpressing KR cells.** KR cells were co-transfected by HA-BEX1 together with the control shRNA (Panel A) or the shRNA for BAX knockdown (Panel B). Forty-eight hours after transfection, 2 µM imatinib was added to the culture medium for 24 hours. Then, KR cells were double stained with annexin V (AV) conjugated FITC and PI.
(TIF)

Table S1 **Sequences of PCR primers for genes clone.**
(DOC)
