# Supplementary figures and images for: BEX1 Promotes Imatinib-Induced Apoptosis by Binding to and Antagonizing BCL-2
Source: PLoS One. 2014 Mar 13;9(3):e91782. doi: 10.1371/journal.pone.0091782 (PMC3953594; doi:10.1371/journal.pone.0091782)

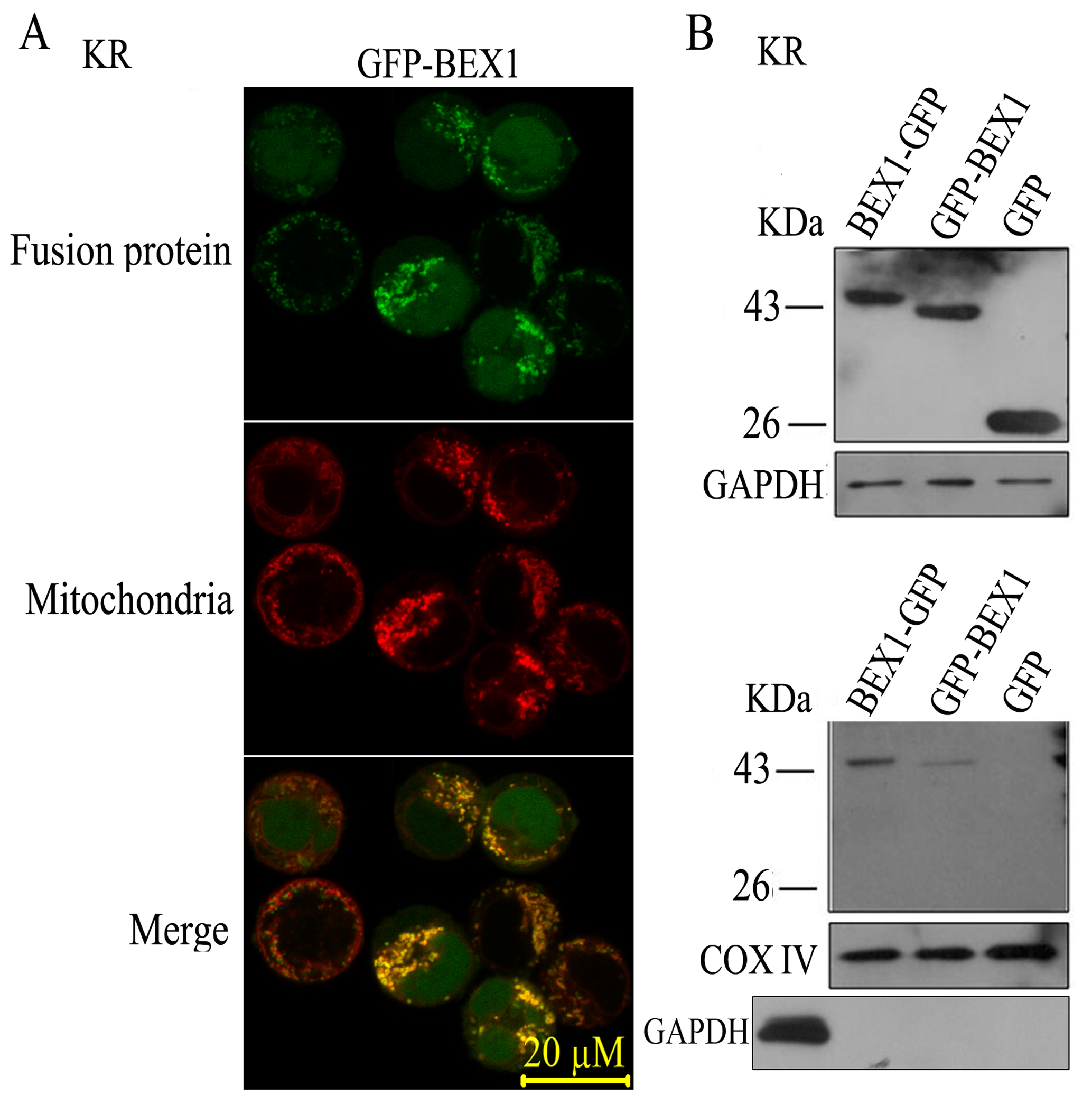

Supplement: Figure S1 — GFP-BEX1 localizes to the mitochondria. A, Fluorescence of live KR cells expressing GFP-BEX1. Cells were visualized for GFP (top), Mitotracker (middle), or merged images (bottom). B, Biochemical fractionation. WCE prepared from KR cells expressing BEX1-GFP, GFP-BEX1 or an empty vector control (GFP) were separated into cytoplasmic (top) and mitochondrial (bottom) fractions and then immunoblotted for GFP, GAPDH, or COX IV. BEX1-GFP showed a better localization to mitochondria (Figure 2A) than GFP-BEX1. Consistently, biochemical fractionation also showed that more BEX1-GFP localizes to the mitochondrial fraction than GFP-BEX1. Also, BEX1-GFP had a larger molecular weight than GFP-BEX1. Although the exact reason for the difference in molecular weight between BEX1-GFP and GFP-BEX1 is not known, one possible explanation is that the GFP-BEX1 visualized on the Western blot may be a degraded form of the protein. This may also explain why less GFP-BEX1 is localized to the mitochondria. (TIF) [file pone.0091782.s001.tif]

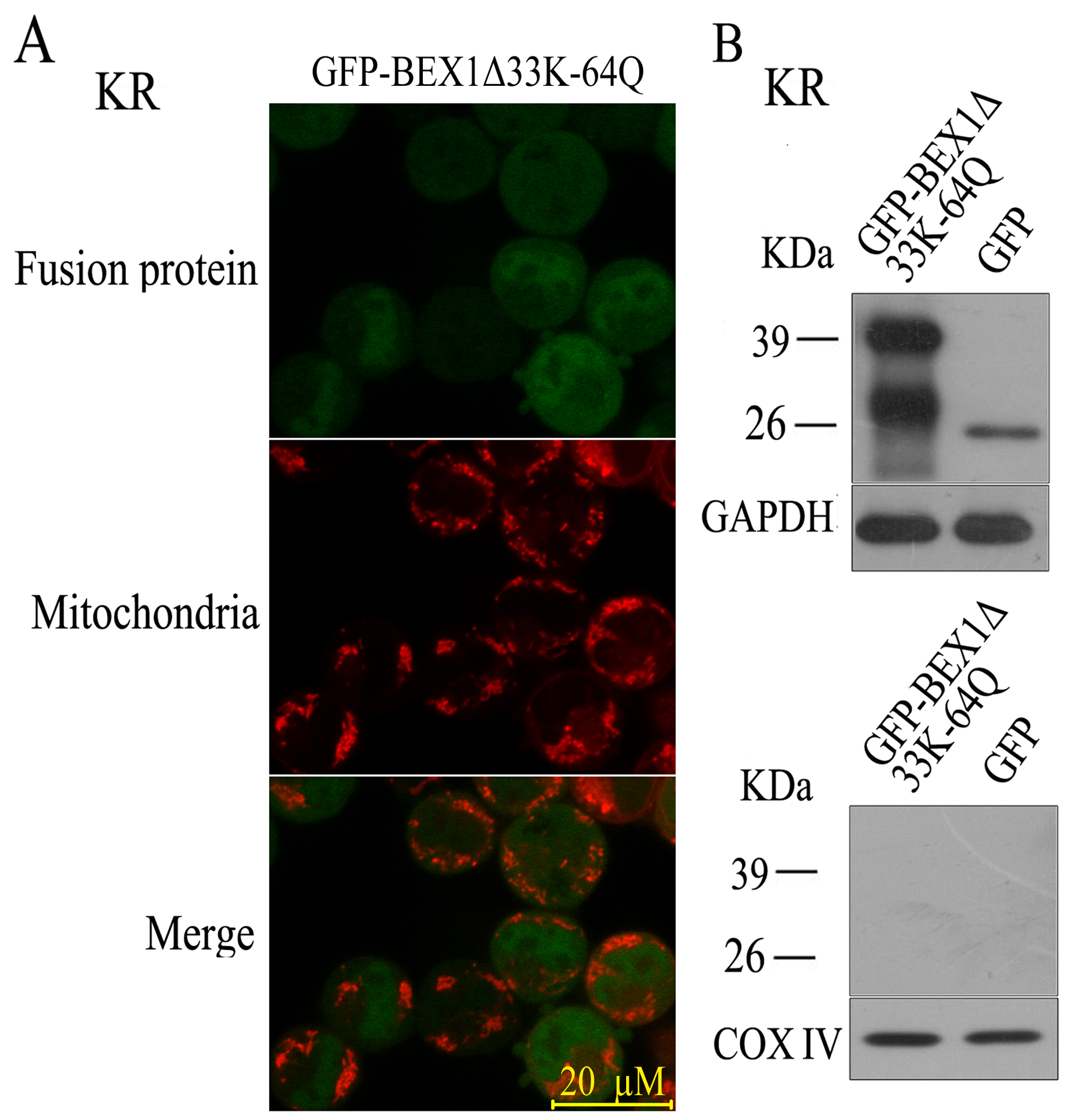

Supplement: Figure S2 — GFP-BEX1Δ33K-64Q fails to localize to the mitochondria without residues 33K-64Q. A. Fluorescence of live KR cells expressing GFP-BEX1Δ33K-64Q. Cells were visualized for GFP (top), Mitotracker (middle), or merged images (bottom). B, Biochemical fractionation. WCE prepared from KR cells expressing GFP-BEX1Δ33K-64Q or an empty vector control (GFP) were separated into cytoplasmic (top) and mitochondrial (bottom) fractions and then immunoblotted for GFP, GAPDH, or COX IV. The bands between 39 kDa and 26 kDa were non-specific signals. (TIF) [file pone.0091782.s002.tif]

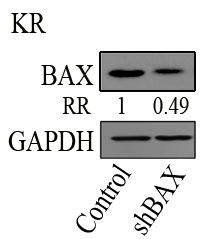

Supplement: Figure S3 — BAX expression in KR cells following shRNA knockdown of BAX. BAX expression was quantified using ImageJ software and was shown as the relative ratio (RR) compared to control shRNA (control) transfected cells. The knockdown efficiency of BAX expression was achieved using shRNA targeting BAX (shBAX), which decreased expression by approximately 50%. (TIF) [file pone.0091782.s003.tif]

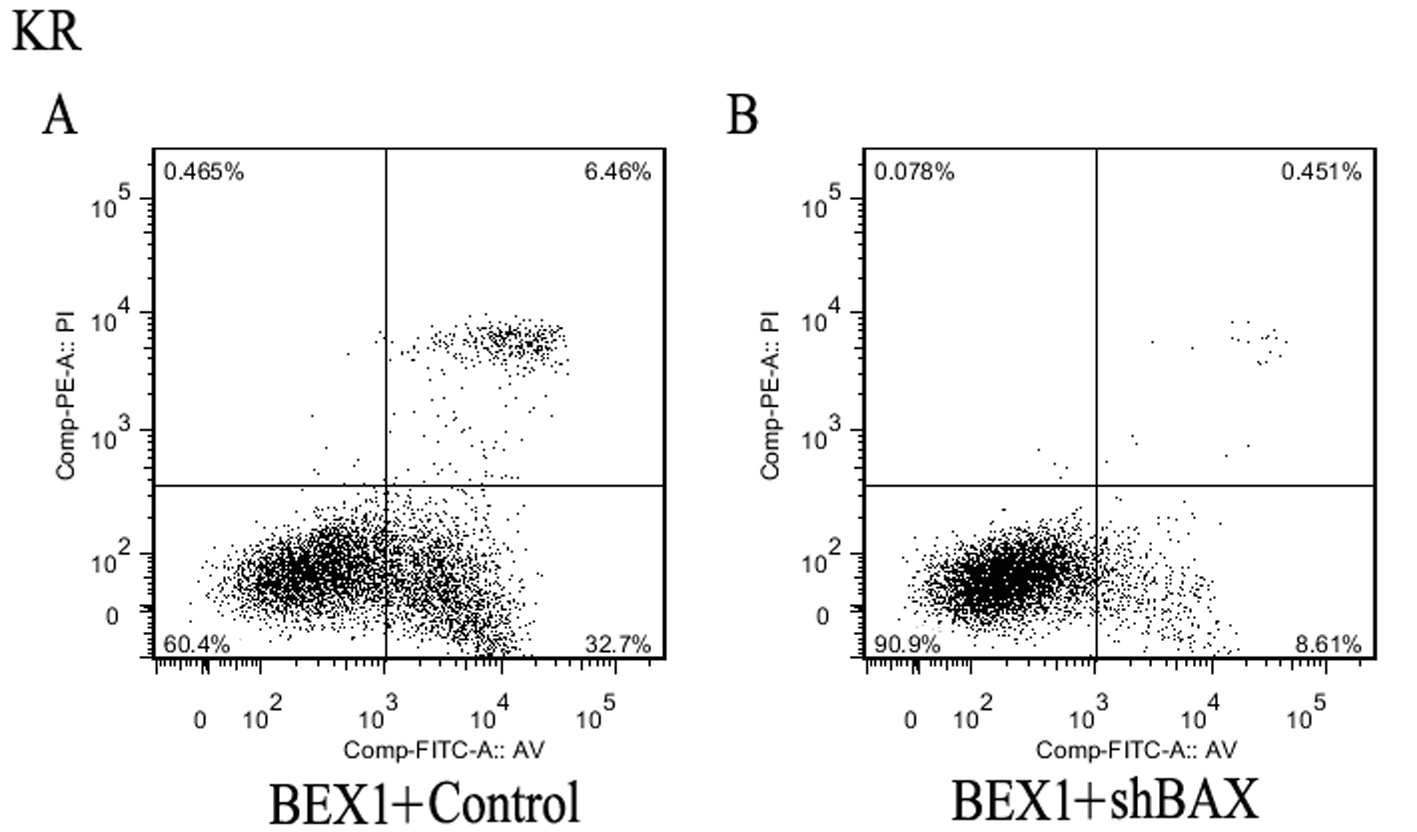

Supplement: Figure S4 — Knockdown of BAX suppresses imatinib-induced apoptosis in BEX1 overexpressing KR cells. KR cells were co-transfected by HA-BEX1 together with the control shRNA (Panel A) or the shRNA for BAX knockdown (Panel B). Forty-eight hours after transfection, 2 µM imatinib was added to the culture medium for 24 hours. Then, KR cells were double stained with annexin V (AV) conjugated FITC and PI. (TIF) [file pone.0091782.s004.tif]
