# Supplementary material for: BEX1 Promotes Imatinib-Induced Apoptosis by Binding to and Antagonizing BCL-2
Source: PLoS One. 2014 Mar 13;9(3):e91782. doi: 10.1371/journal.pone.0091782 (PMC3953594; doi:10.1371/journal.pone.0091782)
Supplement: Table S1 — Sequences of PCR primers for genes clone. (DOC) [file pone.0091782.s005.doc]

Table S1.

| pGBKT7/*BEX1* | 5′-GGAGTAGAATTCATGGAGTCCAAAGAGGAACG-3′; |
| --- | --- |
|  | 5′-TCTGCTGGATCCTCAGGGCATAAGGCAAAACTC-3′ |
| pCMV-HA/*BEX1* | 5′-GGAGTAGAATTCGGATGGAGTCCAAAGAGGAACG-3′ |
|  | 5′-TCTGCTCTCGAGTCAGGGCATAAGGCAAAACTC-3′ |
| pCMV-HA/*BEX1*_1-64_ | 5′-GGAGTAGAATTCGGATGGAGTCCAAAGAGGAACG-3′ |
|  | 5′-TATAATCTCGAGTCACTGCAGGATGGGCTGCCTAA-3′ |
| pCMV-HA/*BEX1*_1-96_ | 5′-GGAGTAGAATTCGGATGGAGTCCAAAGAGGAACG-3′ |
|  | 5′-TAAGACACTCGAGTCACATCAGCTGTCTCACCTCCT-3′ |
| pCMV-HA/*BEX1*_65-128_ | 5′-GCGGTCGAATTCGGTATAGATGGGACATAATGCA-3′ |
|  | 5′-GATAATCTCGAGTCAGGGCATAAGGCAAAACTC-3′ |
| pCMV-HA/*BEX1*_33-96_ | 5′-TAATCATGAATTCGGAAAGGGGAGCCCTTGGCCCTA-3′ |
|  | 5′-TAAGACACTCGAGTCACATCAGCTGTCTCACCTCCT-3′ |
| pCMV-HA*/BEX1*_33-128_ | 5′-TAATCATGAATTCGGAAAGGGGAGCCCTTGGCCCTA-3′ |
|  | 5′-GATAATCTCGAGTCAGGGCATAAGGCAAAACTC-3′ |
| pEGFP-C1/*BEX1* | 5′-GATGTACTCGAGCTATGGAGTCCAAAGAGGAACG-3′ |
|  | 5′-CGATGCTGAATTCTCAGGGCATAAGGCAAAACTC-3′ |
| pEGFP-N1/*BEX1* | 5′-GGAGTACTCGAGGATATGGAGTCCAAAGAGGAACG-3′ |
|  | 5′-TCTGCTGAATTCGCCAGGGCATAAGGCAAAACTC-3′ |
| CDS III /6 Primer | 5'-ATTCTAGAGGCCGAGGCGGCCGACATG-NNNNNN-3' |
| SMART III Primer | 5'-AAGCAGTGGTATCAACGCAGAGTGGCCATTATGGCCGGG-3' |
| ds cDNA Amplify Primer | 5'-TTCCACCCAAGCAGTGGTATCAACGCAGAGTGG-3' |
|  | 5'-GTATCGATGCCCACCCTCTAGAGGCCGAGGCGGCCGACA-3' |
